# Fang Filling, and Treatment of the Alveolar Abscess

**Published:** 1860-08

**Authors:** B. A. Rose


### FANG-FILLING, AND TREATMENT OF THE ALVEOLAR ABSCESS.

BY DR. B. A. ROSE.

Read at a meeting of the Mad River Dental Society.

FANG-FILLING is one among the most important points in operative Dentistry. No half way treatment will answer in such cases. The following method I have found most successful.

When, on examination, we find the pulp exposed, and have no hopes of restoring it and the tooth to a healthy condition, by proper treatment and filling, we make an application of arsenic, morphine and creosote to destroy it. We let this application remain from six to twelve hours, and then remove it and wash out the cavity. Then, with excavators, we enlarge the opening to the pulp cavity, and take a broach dipped in creosote and, with a quick motion, insert it as far as possible into the nerve canal, giving it a rotary motion as it is withdrawn, by which means the greater part of the nerve will be brought with it. This produces a sharp, stinging pain, but is so quickly done that the patient seldom complains of it. The rest of the nerve is to be removed with still smaller sized broaches, dipped in creosote. Then, after washing out the cavity with a syringe and tepid water, take spool thread, about No. 40, saturated with creosote, and fill the nerve cavity, and fill the crown cavity over it with gutta percha. Let the tooth remain thus from five to ten days, when, if no trouble presents itself, remove the temporary fillings, and fill with gold.

For filling fangs, I roll my gold very hard and fine, to correspond with the size of the broach used in cleaning out the canal, and saturate the first pieces with creosote, before introducing them.

Now, as gold is a good conductor of heat, we do not fill the nerve cavity quite full of gold, but soften a small piece of gutta percha and introduce it so as to cut off the connection between the nerve and the crown cavities.

Now, to be successful in fang-filling, there must be space sufficient to enable the operator to have a fair view of the pulp cavity.

TREATMENT OF ABSCESS.—We first ascertain if there is an opening from the crown to the pulp cavity; and if there is not, we make one, and remove all dead and offensive matter from the nerve cavity, and syringe it with tepid water.

Now, if there is a discharge through the alveolar process, enlarge the opening with a lancet, which should penetrate to the apex of the root. We then put an instrument into the nerve cavity, and fill around it with gutta percha. When the instrument is removed, an opening is left for the insertion of the point of the syringe. The canal is now to be injected with creosote, which, if the abscess be of long standing, will pass through the process, along the opening made by the lancet. The gutta percha should then be removed, and the cavity filled with thread, saturated with creosote and chloroform. A piece of lint, moistened with creosote, should be inserted into the incision made with the lancet. This should be continued, and repeated, till there is no more discharge, when the incision in the gum should be allowed to heal. The thread should be removed from the nerve cavity, (as the chloroform and creosote evaporate,) and replaced about once a week, till there is no soreness in the tooth or gums, when it may be filled as safely as in ordinary cases of fang-filling.

